# Salicylic Acid in Rheumatism

**Published:** 1876-03

**Authors:** Charles P. Putnam


Salicylic Acid in Acute Rheumatism.—*By Charles P. Putnam, M. D.*—The Journal for February 10th refers to the lately published cases of rapid recovery from acute polyarthritic rheumatism with salicylic acid. The following case is quite as striking as Traube's.

Alice B., five years old, was thoroughly chilled while skating on February 5th, and for several days after had a hot skin, a coated tongue, and no appetite. On the 9th she lay on the bed most of the day. On the 10th she remained in bed. On the 11th, in the morning, her pulse was 120, her temperature (axillary) 102.7°. In the evening, pulse 131, temperature 103.5. During the latter part of this day she complained much of pain in the muscles of the thigh, in the groin, and in the ankles. She would not move her lower extremities, and could be moved only with great pain. Skin dry. No swelling nor redness of any joints or of other parts of the body. On the 12th the general state was as before; pulse 120, temperature 103. During the day the hands became painful and sensitive, and the knuckle-joint of the third right-hand finger was slightly swollen and rose-colored.

On the 13th, pulse 130, temperature 102.7. She had slept somewhat, with five grains of Dover's powder, but the pain and tenderness of the extremities had increased very much, so that the patient lay quite helpless, cried out when touched, and was moved only with the greatest difficulty. Most of the joints of the hands and feet, especially the joints between the first and second phalanges of the right hand and foot, were swollen and rose-colored. Movement of the jaw also caused pain.

Salicylic acid was prescribed, five grains in wafers every hour when the patient was awake. The treatment was begun at two P. M., and eleven doses, making almost one drachm, had been given at ten A. M., next day. No tinnitus aurium was observed by the patient. After the first three doses there seemed to be some improvement, for though the physical appearances remained unchanged, the patient's spirits were better.

After the seventh dose she went to sleep, and was quiet most of the night, only waking often enough to take two more doses. In the morning she turned over in bed, moved the bedclothes with her hands, and wanted to be dressed.

On examination it was found that the redness had left all the joints; they could be moved without pain, and only a moderate œdematous swelling marked the affected parts. The patient complained only of slight itching of the hands and face.

At ten A. M., pulse 120, temperature 101.6. At six P. M., pulse 124, temperature 102.4. Five grains of salicylic acid were ordered every two hours during the night, but only three doses were given, as the patient seemed quite comfortable, and slept. On the 15th (forty-eight hours after treatment began) the temperature was 99.9.—*Boston Medical and Surgical Journal.*

